# Donor/recipient enhancement of memory in rat hippocampus

**DOI:** 10.3389/fnsys.2013.00120

**Published:** 2013-12-26

**Authors:** Sam A. Deadwyler, Theodore W. Berger, Andrew J. Sweatt, Dong Song, Rosa H. M. Chan, Ioan Opris, Greg A. Gerhardt, Vasilis Z. Marmarelis, Robert E. Hampson

**Affiliations:** ^1^Department of Physiology and Pharmacology, Wake Forest School of MedicineWinston-Salem, NC, USA; ^2^Department of Biomedical Engineering, University of Southern CaliforniaLos Angeles, CA, USA; ^3^Department of Neurobiology, Chandler Medical School, University of KentuckyLexington, KY, USA

**Keywords:** memory-transfer, rodent, ensemble, non-linear model, electrical stimulation

## Abstract

The critical role of the mammalian hippocampus in the formation, translation and retrieval of memory has been documented over many decades. There are many theories of how the hippocampus operates to encode events and a precise mechanism was recently identified in rats performing a short-term memory task which demonstrated that successful information encoding was promoted via specific patterns of activity generated within ensembles of hippocampal neurons. In the study presented here, these “representations” were extracted via a customized non-linear multi-input multi-output (MIMO) mathematical model which allowed prediction of successful performance on specific trials within the testing session. A unique feature of this characterization was demonstrated when successful information encoding patterns were derived online from well-trained “*donor*” animals during difficult long-delay trials and delivered via online electrical stimulation to synchronously tested naïve “*recipient*” animals never before exposed to the delay feature of the task. By transferring such model-derived trained (*donor*) animal hippocampal firing patterns via stimulation to coupled naïve *recipient* animals, their task performance was facilitated in a direct “*donor-recipient*” manner. This provides the basis for utilizing extracted appropriate neural information from one brain to induce, recover, or enhance memory related processing in the brain of another subject.

## Introduction

To understand the neural basis of memory, several features of the context in which the memories occur and are utilized, and the functional aspects of the brain areas involved, need to be identified and controlled (Hampson et al., 2008; Eichenbaum and Fortin, 2009). In prior studies we achieved both of these important contingencies as well as (1) overcoming possible alternative interpretations of the relationship between recorded hippocampal ensemble activity and the behavioral task in which short-term memory formation is necessary (Deadwyler and Hampson, 2006; Deadwyler et al., 2007), and (2) developing an effective mathematical/operational model for online prediction of CA1 hippocampal cell activity from simultaneously recorded input firing patterns from synaptically connected CA3 neurons (Song et al., 2009; Berger et al., 2011; Hampson et al., 2011). The combination of these approaches was made possible by the chronic recording of neural firing patterns in the above two major hippocampal subfields via specially designed mutineuron recording arrays that allowed simultaneous detection and analysis of behaviorally critical ensemble discharge patterns (Deadwyler and Hampson, 1997; Hampson et al., 1999, 2008). It has been shown that a non-linear multi-input/multi-output (MIMO) mathematical model provides the mean to translate the above ensemble activity into a format that allows predictions of CA1 firing patterns from CA3 activity required for successful task performance (Marmarelis, 2004; Zanos et al., 2008; Song et al., 2009).

In the following paper we demonstrate a critically important feature of the online extracted firing patterns of hippocampal ensembles by showing how alteration and facilitation of hippocampal function can be employed via direct connection with the same structure in a different “*donor*” animal performing the same task at the same time. A recent study (Pais-Viera et al., 2013) reported similar brain-to-brain transfer by directly stimulating motor cortex in the brain of a recipient rodent from a different animal. However, we report here the discovery that appropriate neural firing patterns which encode useable *memory* can be derived online from trained animals, and inserted via electrical stimulation of those same hippocampal regions to animals untrained to perform the memory extended requirement of the delayed-non-match-to-sample (DNMS) task. These findings confirm the functional significance of a previously identified “hippocampal prosthesis” (Berger et al., 2011) shown to repair and/or enhance damaged or disrupted memory processes in the same animal. However, the outcomes of the study described here also indicate ways of using brain systems from non-impaired subjects to impart functional information when and where it did not get formulated in affected subjects, via a *donor-recipient* paradigm.

## Methods

### Information encoding by hippocampal neural ensembles during performance of a delayed-non-match-to-sample (DNMS) memory task

**Figure 1:**
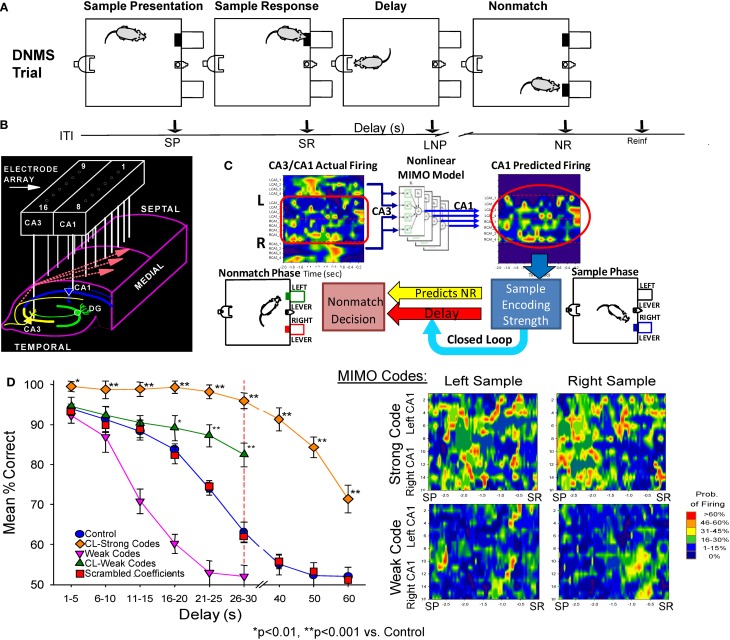
**Delayed non-match to sample (DNMS) task, MIMO model and associated hippocampal ensemble activity. (A)** *DNMS Trial Diagram*. Sample lever presentation (SP) and Sample response (SR) are followed by a variable delay interval which required a nosepoke (NP) in a photocell on opposite wall. The Non-match phase began after delay timeout, with both levers presented simultaneously for reward contingent Non-match Response (NR) on the lever opposite the SR position. Correct non-match responses produced 0.2 ml of water delivered to the trough between the levers. Timeline below shows sequence of task phases: ITI–intertrial interval; SP–Sample Lever presentation; SR–Sample response; Delay–Delay interval; LNP–last required nosepoke during Delay; NR–Non-match (decision) response; Reinf.—Delivery of water reward. **(B)** Hippocampal recording array: two rows of 8 stainless steel 20 μm wires positioned longitudinally within hippocampus at 200 μm intervals for each electrode pair in CA3 and CA1 cell layers. Arrays were implanted bilaterally in both hippocampi providing a total of 32 indwelling chronic electrodes per animal. **(C)** Heatmap display (left) showing online array monitored hippocampal ensemble single neuron (actual firing) activity. Low-to-high (blue-to-red) firing rates are indicated at the separate CA3/CA1 locations on the array **(B)** during the occurrence of the SR (time 0.0 s). Schematic of non-linear MIMO model: Spike trains X_1_–X_8_ recorded from CA3 electrodes (CA3 input) on the hippocampal array (left) are input to the model and used to predict CA1 firing across the other 8 recording locations shown in the diagram on the right (1–8, predicted CA1) at the time of the SR. The schematic of the non-linear analysis used to construct the CA1 predicted outputs which illustrates estimation of the spatiotemporal relationship between each CA1 output (Y) and multiple CA3 inputs (X) modeled via Volterra kernels which are then combined to form the MIMO model for all CA1 locations (see Supplemental Material). The output of the model (right) is then employed to vary the delay interval of the DNMS task on the same trial in a closed loop manner as shown by the diagram below. Lower Right: MIMO Codes: Heatmap displays of MIMO model predicted CA1 firing in both hemispheres during the response on the Sample lever on individual trials during sample presentation (SP and response (SR) for trials both Left and Right sample lever presentation. Strong Codes: MIMO predicted CA1 sample lever firing on *successful trials.* Weak Codes: MIMO prediction of the same CA1 cell firing on *error trials.* Firing rates indicated by the scale bar at right. **(D)** MIMO mediated closed loop control of DNMS performance (mean ± s.e.m. % correct) summed over all animals, *n* = 15). Trials in which *strong (diamonds) and weak (triangles) SR codes* occurred are plotted as a function of length of delay, shown compared to Control performance on trials not sorted by code strength. Performance on trials with extended delays (40, 50, or 60 s, vertical dashed line) was significantly higher than on trials with the same delays (Control, 40–60 s) presented without MIMO Closed Loop regulation [*F*_(1, 401)_ = 18.39, *p* < 0.001, ^*^p < 0.01, ^**^p < 0.001, Closed Loop vs. Control trials]. DNMS (performance) for trials of 1–30 s delay (Control) is also shown compared to performance on trials in which only *weak SR codes* (Weak Codes) occurred [*F*_(1, 401)_ = 11.81, *p* < 0.001]. Performance on trials in which the MIMO model coefficients were randomly assigned (i.e., scrambled) to CA1 firing are also shown in the curve for scrambled coefficients (squares) as having no difference from Control performance.

Recording and analysis of hippocampal mutineuron activity over a number of years in rodents performing a DNMS memory task (cf. Hampson et al., 2008, 2011) provided the basis for application of a non-linear MIMO model to hippocampal neural ensemble firing patterns in the first demonstration of a memory prostheses in rodent brain (Berger et al., 2011; Hampson et al., 2012a,b). This extensively studied DNMS task requires rats to retain the position of a “Sample” lever that is presented and responded to (i.e., sample response: SR) at the start of the trial, over a temporal delay interval of variable duration (1–30 s) in order to make a “Non-match” response (NR) on the lever in the opposite position when both levers are presented simultaneously at the end of the delay (Figure 1A). During the delay period a nosepoke into a photocell on the wall opposite the levers is required to proceed to the Non-match phase. If there is no delay a single nosepoke produces both levers, for delays of increased duration a single nose poke is still the only requirement but animals make multiple nosepokes until the light terminates above the photocell as an indicator of delay termination and both levers are presented on the opposite wall (Figure 1A). Figure 1D (control) shows that DNMS performance accuracy decreases linearly as a direct function of the duration of the interposed delay interval.

### Non-linear multi-input multi-output (MIMO) model detection and prediction of hippocampal ensemble memory codes

Electrophysiological recording during the DNMS task employs custom designed arrays of microwire (20 μm) electrodes implanted bilaterally in the hippocampus in each hemisphere to provide single neuron firing data from 8 pairs of aligned CA3-CA1 probes arranged at 200 μm intervals along the longitudinal axis in the dorsal hippocampus in rodent brain (Figure 1B). The neural correlates obtained from studies with these techniques in the DNMS task have been utilized in more than 2000 animals with respect to type and amount of behavioral training required for maximal performance in conjunction with extraction of distinct patterns of neural ensemble activity correlated with successful performance (Deadwyler and Hampson, 2006; Hampson et al., 2008, 2011). In recent studies the trial-by-trial nature of changes in ensemble firing patterns has been described in relation to non-linear fluctuations associated with successful task performance (Figure 1C), as well as applying the same non-linear model for reversing detrimental actions of drugs on performance (Song et al., 2009; Berger et al., 2011; Marmarelis et al., 2013).

This very precise MIMO non-linear mathematical model (Figure 1C) was employed to determine the “strength” of ensemble SR firing patterns or “codes” formulated specifically on successful (strong code) or error (weak code) DNMS trials (Figures 1C,D) across all delay durations. The application of this model (see *Supporting Material*) allowed prediction of CA1 neuron firing “output” patterns based on the “input” to the model (CA3 neuron firing) using Laguerre expansions of Volterra Kernels to determine the temporal relationships between spike occurrences recorded in these two areas during the task (Song et al., 2009, 2013; Berger et al., 2012). As shown previously the inputs to the MIMO model were CA3 cell discharges associated exclusively with outputs from simultaneously recorded postsynaptic CA1 cells connected via Schaeffer collateral monosynaptic connections (Witter and Amaral, 2004). Hence, as shown at the lower right in Figure 1C, the MIMO model analysis of CA3 and CA1 spike occurrences associated with critical DNMS task events provided the basis for online “detection” (CA3) and “prediction” of CA1 firing patterns associated with successful (strong code) vs. error (weak code) trials (Berger et al., 2011; Hampson et al., 2011). As a final verification that the MIMO model output could predict behaviorally relevant hippocampal encoded information, online calculations were utilized in a closed loop paradigm in which the detection of strong SR codes (Figure 1C) was used to adjust the difficulty of the same trial via increased or decreased delay duration. In accordance with the strength of SR codes (Figure 1D) performance was either above or below that on trials in which such SR codes were not present (Hampson et al., 2012a,b). This closed loop procedure served as the basis for the next phase in which strong SR code patterns were detected, mimicked and then administered as electrical stimulation, which is described next.

### External insertion of MIMO derived firing patterns via electrical stimulation of hippocampal CA1 neurons

**Figure 2:**
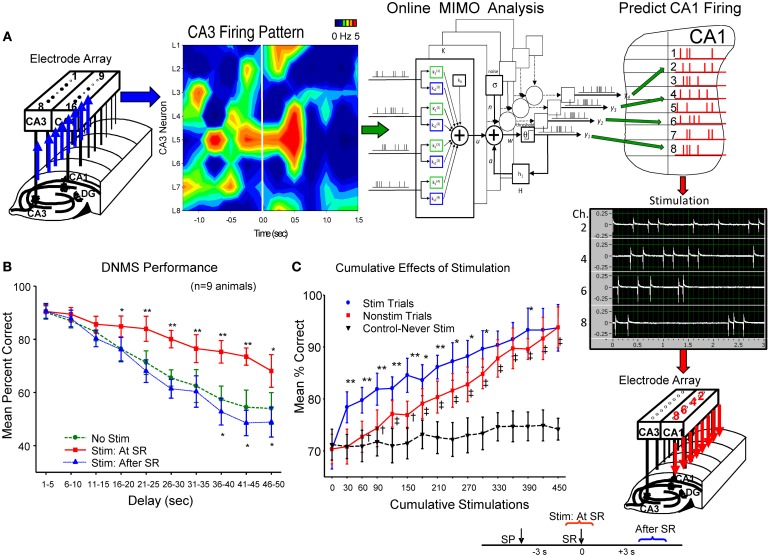
**Electrical stimulation utilizing MIMO predicted CA1 output patterns, facilitates DNMS performance. (A)** Patterns of recorded CA3 cell firing in hippocampal array, shown as a heatmap (left), constitutes the input for online implementation of the MIMO model (center) to predict CA1 firing pattern (Figure 1C) indicated by red “tick” marks in hippocampal (CA1) layout (at right). This MIMO output pattern is fed to a programmable 8 channel stimulator (Supplemental Material) which delivers up to 3.0 s trains of bipolar electrical stimulation pulses (middle right) to the CA1 electrode locations showing the same firing pattern in each hemisphere. Stimulator output (photo display) is shown for 4 of the 8 channels to indicate different frequencies and intensities of stimulus trains delivered to separate CA1 locations (Supplemental Material). The time lag between CA3 recording, MIMO calculation and output of CA1 stimulation was approximately 50 ms. **(B)** DNMS performance graph of trained animals (*n* = 9) for delays of 1–60 s compares effects of 3.0 s stimulation delivered either: (1) at the time the SR occurred (Stim at SR) vs. No Stim [*F*_(1, 731)_ = 11.50, *p* < 0.001], or (2) delayed for 3.0 s after the SR was made (Stim after SR) vs. No Stim [*F*_(1, 731)_ = 3.17, n.s.] (see inset lower right). Asterisks (^*^p < 0.01, ^**^p < 0.001) indicate significant difference in DNMS performance compared to control (No Stim.) trials (Berger et al., 2011). **(C)** Cumulative effects of MIMO generated SR stimulation over successive trials (Hampson et al., 2012a) shows progressive increase in overall mean (± s.e.m.) % correct performance in 30 trial blocks for animals (*n* = 5) receiving 25–30 SR stimulation trials (Stim Trials) per session for 15 sessions. Red curve (squares) shows overall performance on remaining trials within the same behavioral sessions in which no stimulation was delivered (No Stim). Inverted triangles (dotted line) shows performance over the same number of successive trials of equivalently trained animals (*n* = 20) that never received SR stimulation (Never Stim). Stim vs. Non-stim trials: *F*_(1, 145)_ = 9.42, ^*^p < 0.01,^**^p < 0.001, Stim. vs. Never Stim: *F*_(1, 1349)_ = 15.72, *p* < 0.001, Non-stim vs. Never Stim. *F*_(1, 1349)_ = 11.29, ^†^*p* < 0.01, ^‡^*p* < 0.001.

The above successful application of the MIMO model provided the unique basis for activating CA1 cells if model derived inputs from CA3 neurons were no longer operative (Berger et al., 2011). This was accomplished by transforming the CA1 cell output pattern of the MIMO model into trains of electrical stimulation pulses (1.0 ms biphasic 20–100 μA) and delivering them in real time to the same CA1 electrode locations in the recording arrays via a multichannel stimulator (Figure 2A). CA1 stimulation patterns were therefore similar to the strong code SR firing patterns derived from each animal by the MIMO model from the same CA1 electrodes. Stimulation was delivered online via the inputs detected from CA3 electrodes on the same hippocampal array as shown in Figure 2A for hippocampal implants in both hemispheres. Intensities of stimulus pulses (20–100 μA) delivered to CA1 were adjusted to provide indications of extracellular current flow (i.e., local field potentials) at adjacent CA1 electrode locations on the same array (see below). MIMO generated strong code CA1 SR stimulation pulse trains were of 3.0 s duration and delivered within 50 ms of the detection of corresponding input patterns recorded in CA3 (see *Supporting Material*). Since both the pattern and time of application of the strong code CA1 SR stimulation were related directly to the MIMO model detection of corresponding CA3 input firing, it was also possible to deliver CA1 SR stimulation on trials in which “strong code” input patterns were not detected in CA3 recordings. This provided the means to facilitate performance above control levels by delivering strong code CA1 SR stimulation on trials that normally did not generate strong codes in CA3 or CA1 naturally (Berger et al., 2011; Hampson et al., 2012a,b).

The effectiveness of SR CA1 stimulation patterns is shown in Figures 2B,C as marked increases in DNMS task performance on stimulation trials in comparison to trials in which no stimulation was delivered (No Stim). To control for other possible actions, the specificity of the CA1 stimulation pattern with respect to encoding of the SR was tested directly by delaying delivery of the same stimulation pattern to CA1 until 3.0 s after the SR which as shown in Figure 2B (Stim after SR) produced no changes in performance from control (No Stim) levels. Further verification was revealed by comparing trials in which SR CA1 stimulation was generated from different MIMO firing patterns with “scrambled” coefficients between neurons (Figure 1D) which actually impaired performance in some cases as shown in other studies (Hampson et al., 2012a). A final test of the similarity of the stimulation patterns to actual CA1 output firing patterns was assessed by repeating the procedure over several sessions and examining the trial-by-trial cumulative effects of continued exposure to MIMO predicted SR CA1 stimulation as shown in Figure 2C (Berger et al., 2011; Hampson et al., 2012b). These procedures verify that functional encoding of the SR could be imposed in subjects performing the DNMS task by matching the MIMO predicted CA1 firing pattern with stimulation pulses delivered within 50 ms to the same CA1 loci (Figure 2A), which provided encoding of lever position necessary to perform the task successfully across all interposed delay intervals.

### A method for transfer of MIMO SR stimulation from *donor* to delay-naïve *recipient* animals

**Figure 3:**
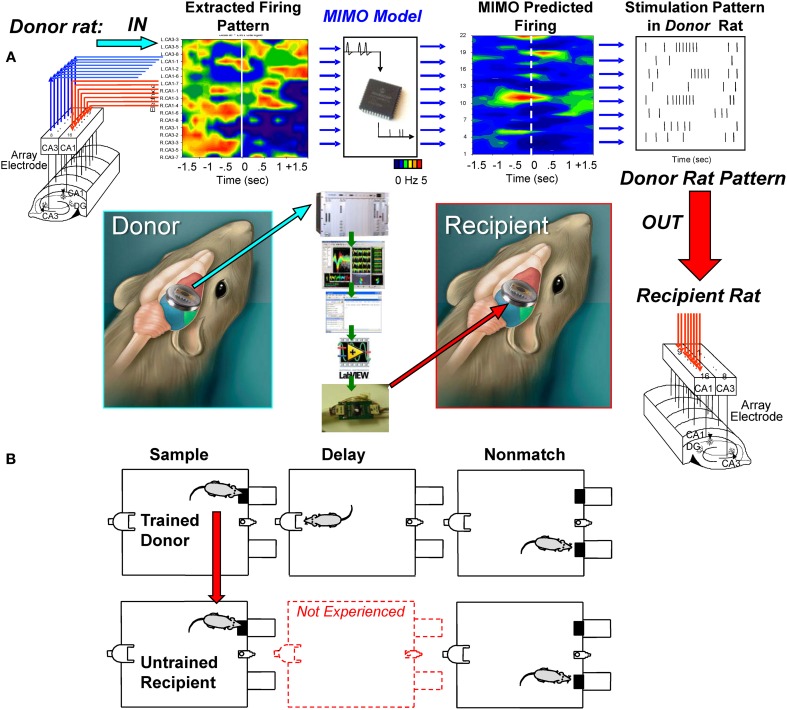
**Transference of successful MIMO coded ensemble firing patterns from trained “*donor*” rats to task-naïve “*recipient*” rats. (A)** Recordings were obtained online from well-trained animals (i.e., *donor rats*) with validated effective MIMO SR CA1 stimulation patterns as shown in Figure 2. A second group of delay-naïve animals (*recipient rats*) were only trained to perform the operant responses in the DNMS task in sequence without exposure to variable and extended delay intervals interposed between the SR and NR task phases requiring completion of the nosepoke response on the opposite wall (red middle diagram). **(B)** *Donor-Recipient rat* “pairs” were recorded from and tested simultaneously in different chambers with DNMS trial execution synchronized by presentation of the sample lever in the same position at the same time. During performance of trials within the simultaneous sessions, *donor rat* hippocampal ensemble activity was monitored for presence of CA3 firing predictions of effective *strong SR code* CA1 stimulation patterns (Figure 2). When such *donor rat* strong code patterns occurred, the associated MIMO-predicted SR CA1 stimulation pattern was routed instead to the CA1 electrodes in the *recipient rat* hippocampus while performing the *SR* within 1–3 s after detection of *donor rat* strong SR code. Delay intervals of 8, 12, or 16s were then introduced on the same trial for the *recipient rat* which required the previously learned selection of the opposite lever in the Non-match phase of the task after timeout of the unfamiliar delay periods. All trials on which delays were imposed to *recipient rat*s were determined when strong SR codes were generated by *donor rats*; hence occurrence of all delay trials during *recipient rat* sessions was essentially random and unpredictable.

The above MIMO model SR CA1 stimulation method served as the basis for testing a unique dual animal “*donor/recipient*” paradigm in which (1) a well-trained “*donor rat*” performed the DNMS task in one chamber at the same time as (2) a delay-naïve “*recipient rat*” was tested at the same time in a different chamber in a trial synchronized manner (Figure 3A). The “*recipient rat*” was not trained to perform the DNMS task over intervening delay intervals >1.0–3.0 s (red dotted delay phase in Figure 3B) which was the time it took to make the required nosepoke response on the opposite wall of the chamber to present the Non-match phase (Figure 1A). The imposition of trials with extended delay intervals during the session constituted the first exposure of *recipient rats* to the task requirement for retention of SR information across increased time intervals (8–16 s) in order to correctly select the opposite lever in the Non-match phase (Hampson et al., 2008). Performance in the Sample phase of the task was synchronized between animals by presentation of the Sample lever in the same position at the same time in both chambers to initiate the same trial simultaneously for both animals (Figure 3B). On synchronous trials in which the MIMO model applied to the *donor rat* CA3 firing patterns, generated a successful strong code SR CA1 pattern (Figure 2A), the stimulus pulses representing that MIMO strong SR code were routed instead to the corresponding CA1 electrodes in the *recipient rat* (Figure 3A) performing the SR at approximately the same time. For the naïve *recipient rat* following the delivery of the *donor rat* strong code SR CA1 stimulation pattern after the SR, an unfamiliar delay interval of 8, 12, or 16 s was introduced into the trial prior to onset of the Non-match phase. Since routing of SR CA1 stimulation to the *recipient rat* was determined by concurrent CA3 encoding strength in the hippocampus of the simultaneously performing *donor rat*, extended delay trials for the *recipient rat* occurred randomly within the paired sessions. As a control procedure, performance was compared on trials with the same delays administered to *recipient rats* by *donor rat* CA3 encoding strength on the same trial but without delivery of SR CA1 stimulation.

## Results

### Transfer of memory to delay-naïve *recipient* animals by delivery of MIMO stimulation from trained *donor* animals

**Figure 4:**
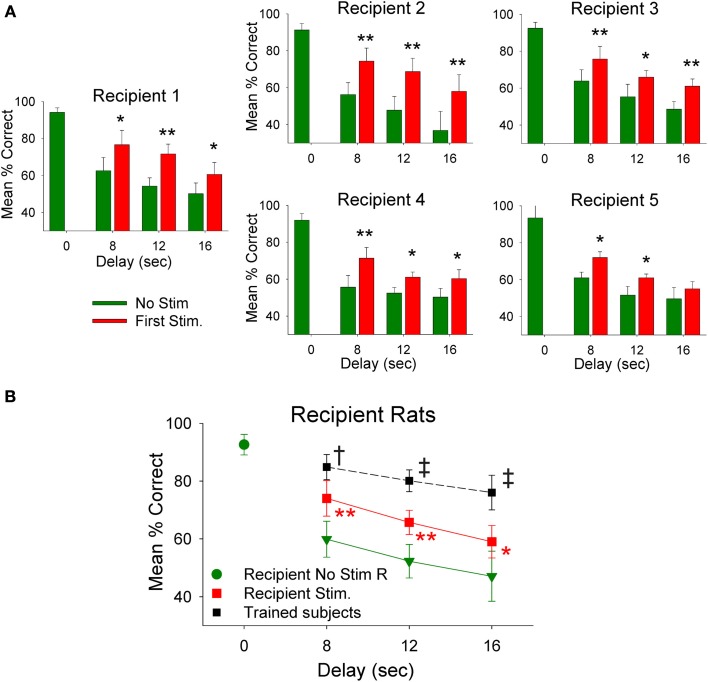
***Recipient rat* performance on DNMS trials with unfamiliar superimposed delays facilitated by *donor rat* mediated SR stimulation. (A)** Individual DNMS performance of five different *recipient rats* subjected to trials with 8, 12, and 16 s delays shown for trials in which no stimulation was delivered (No Stim) or on trials on which a simultaneously paired *donor rat* delivery of MIMO SR CA1 stimulation pattern was delivered (Stim). The similarity across each graph indicates generality of facilitated performance on imposed delay trials with delivery of *donor rat* MIMO generated CA1 SR stimulation. Asterisks ^*^p < 0.01, ^**^p < 0.001, Stim *donor rat* vs. No Stim. **(B)** Overall performance of *recipient rats* (*n* = 5) is shown as mean (±s.e.m.) % correct trials with no delays (green dot−0.0 s values) in comparison to trials with variable delays (8, 12, 16 s) without *donor rat* stimulation (green triangles-No Stim) delivered during the trial [*F*_(3, 279)_ = 3.61, *p* < 0.001]; and performance on trials with the same delays but including *donor rat* MIMO strong SR code stimulation (Recipient Stim, red squares) which significantly improved performance compared to No Stim trials [*F*_(1, 279)_ = 9.82, *p* < 0.001]. For comparison a plot of the average performance level of rats fully trained (*n* = 20) on the task at the same delays (Trained subjects) is shown (black squares) for comparison to *recipient rat* performance on stimulated trials [*F*_(1, 1349)_ = 13.48, *p* = 0.001, Trained subjects > *recipient rats*]. Symbols: ^*^p < 0.01, ^**^p < 0.001, ^†^*p* < 0.01, ^‡^*p* = 0.001.

The employment of *donor rat* MIMO model generated SR CA1 stimulation was applied to test whether it was possible to facilitate performance in *delay-naïve recipient rats* untrained in the delay version of the DNMS task. Results in Figure 4A show the mean % correct performance of 5 different *recipient rats* subjected for the first time to 8, 12, and 16 s interpolated delays with MIMO derived SR CA1 stimulation patterns (Stim) delivered on half the trials from synchronously performing *donor rats. Donor rat* SR CA1 stimulation allowed *recipient rats* to perform significantly better than if they did not receive stimulation (No stim) and these levels are compared with their higher performance on trials in which no delay was interspersed (0.0 s). The overall performance of all *recipient* is shown in Figure 4B for recipient-stim and recipient-no stim trials in which highly significant improved performance is apparent across all delays. Figure 4B also shows that recipient-stim average performance was significantly below that of fully trained animals that did not receive SR stimulation at those same delays (trained subjects, *n* = 23).

**Figure 5:**
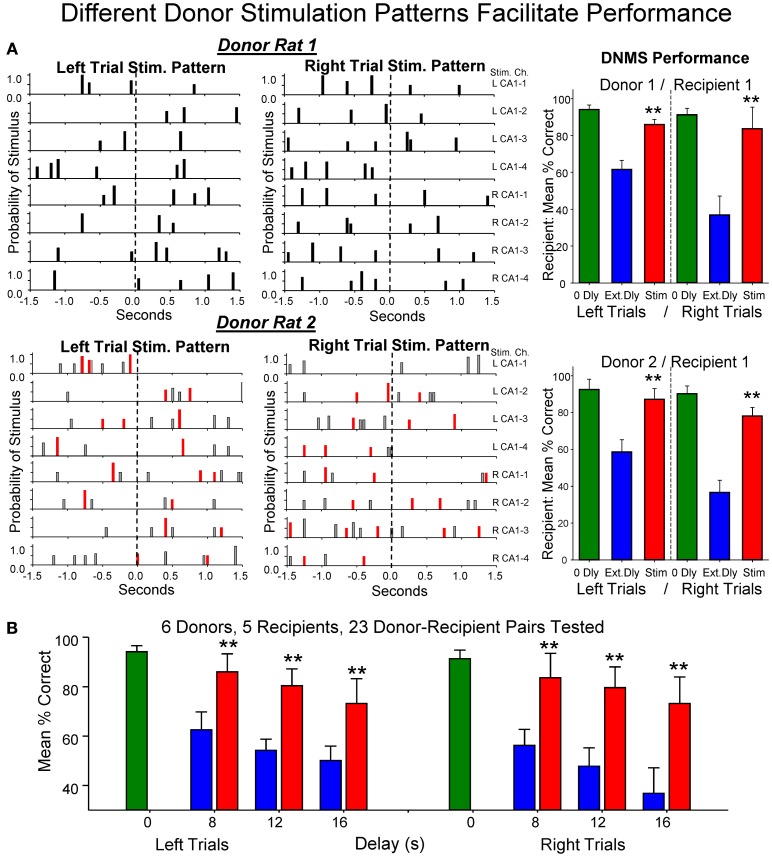
**Enhancement of performance of the same *recipient rat* by different *donor rats*. (A)** Left: Delivered MIMO SR CA1 stimulation patterns showed for left and right lever trials from *Donor rat 1* (upper) and *Donor rat 2* (lower). Red marks in *Donor rat 2* patterns reflect occurrences of identical pulses delivered in *Donor rat 1* pattern (above) for direct comparison of the two SR Stimulation patterns delivered to the same *Recipient rat* on different trials. Right: Overall performance of the same *Recipient rat* for sessions in which SR Stimulation (Stim) on delay trials was contributed by *Donor rat 1* (upper) and *Donor rat 2* (lower) for left and right Sample lever trials summed over all delays (red) compared with delay trials in which SR stimulation was not delivered (blue). Green bars represent performance by the same *Recipient rat* on trials with no delay (0 Dly) presented in the same sessions as described above. **(B)** Lower plot shows overall average performance for all *Donor/Recipient* sessions (*n* = 23) for trials with Left and Right SR position and those which received *donor rat* SR stimulation (red) vs. no stimulation trials (blue) as a function of delay (0, 8, 12, 16 s). Plots include all *Donor/Recipient* pairs, 5 different *recipient rats* paired with one or more *donor rats* (*n* = 6). Asterisks (^**^p < 0.001) indicate significant difference compared to trials with no *donor rat* stimulation (No Stim).

The physiologically specific nature of the *donor rat* stimulation was further verified by the fact that performance was facilitated in a delay dependent manner (Figures 4A,B) in the same way that natural performance was affected by the duration of interposed delays during the trial. This was further verified by comparing the effects of MIMO stimulation patterns delivered by different *donor rats* to the same *recipient rat* within, as well as, across different behavioral sessions. Figure 5A shows the comparison of performance of the same *recipient rat* receiving SR stimulation from two different *donor rats* over similar interposed delay trials. Figure 5A shows the patterns of SR CA1 stimulation delivered by each *donor rat (donor rats 1 and 2)* on both left and right SR lever trials. Although there were slight differences with respect to the spatiotemporal delivery of CA1 stimulation pulses, the overall patterns related to the time of execution of the SR were highly similar. The graphs to the right in Figure 5A show the average performance of the same *recipient rat* on similar types of trials with stimulation and non-stimulated extended delays from the same two *donor rats.* It is clear that stimulation generated by both *donor* rats on different trials in the same session facilitated performance of the *recipient rat* in nearly identical fashion. Figure 5B summarizes the performance of all *recipient rats* (*n* = 5) over the 3 delay intervals for all *donor rat* (*n* = 6) stimulation (Stim) vs. non-stimulated (Non-Stim) trials compared to trials in which no delay was imposed (0 s).

Control manipulations performed to insure that *donor rat* SR stimulation was the basis for the improved performance of *recipient rats* included: (1) changing the *donor rat* stimulation patterns in different ways such as scrambling coefficients (Figure 1A), (2) delivering *donor rat* stimulation patterns at different times (i.e., 3.0 s) after the SR (Figure 2B), and (3) delivery of stimulation based on *recipient rat* MIMO-extracted SR CA1 patterns on the trials with interpolated delays determined by *donor rat* encoding patterns (not shown). None of the latter control procedures produced significant increases in *recipient rat* performance above that exhibited on trials with the same delays and no *donor rat* stimulation (Figure 4 No Stim, Figure 5B).

### Neural basis for MIMO stimulation enhanced memory

**Figure 6:**
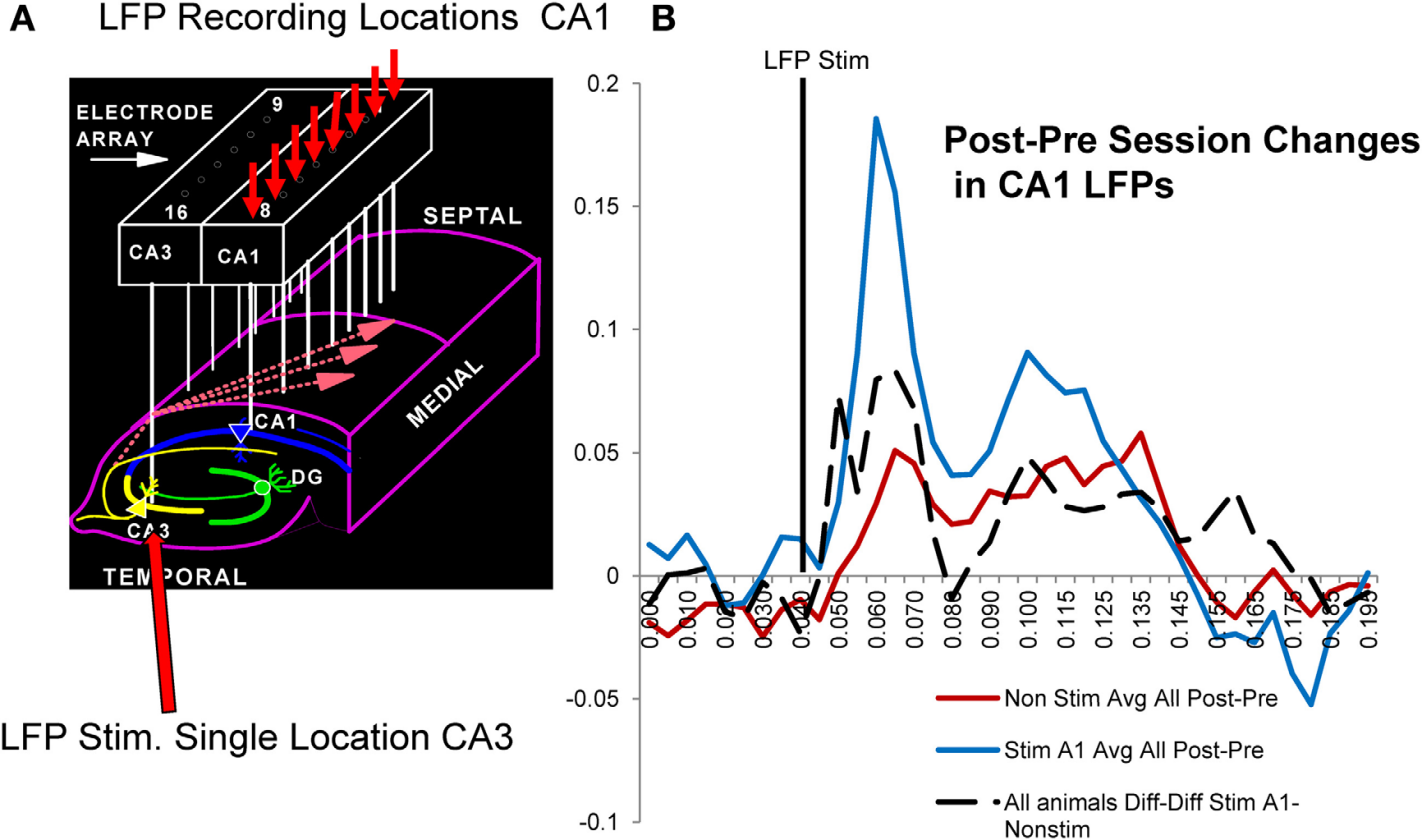
**Possible synaptic basis for facilitative *Donor/Recipient* MIMO SR stimulation. (A)** Illustration of hippocampal synaptic connections between CA3 and CA1 cells in the same hippocampal region occupied by the same electrode array used to deliver SR Stimulation (Figures 1–3). Arrows show divergent projections from a single CA3 cell to multiple CA1 cells via Shaffer collateral connections used to determine changes in CA1 (small red arrows) local field potentials (LFPs) elicited by stimulation delivered to a single CA3 locus (large red arrow) before (Pre) and after (Post) behavioral sessions with MIMO SR Stimulation vs. non-stimulated sessions. **(B)** Average CA1 LFPs elicited by CA3 stimulation are plotted as differences (Post-Pre_diff_) in voltage amplitude measured at the indicated time points (10 ms) of the LFP after stimulus pulse delivery (vertical black line). Red: Mean Post-Pre_diffs_ in LFP amplitudes recorded prior to and following non-stimulation sessions for trained animals. Blue: Average Post-Pre_diffs_ in LFP amplitude following sessions in which SR stimulation was delivered to facilitate performance. Positive Post-Pre_diffs_ reflect average voltage changes related to increased CA1 LFP components after the behavioral session relative to voltages elicited by the same CA3 current intensities prior to the session. These Post-Pre_diffs_ for sessions in which SR stimulation facilitated task performance (blue curve) are shown compared to Post-Pre_diffs_ for those sessions in which stimulation was not delivered (red curve). Dotted: Diff-Diff shows average difference between CA1 LFP Post-Pre_diffs_ for SR stim vs. non-stim sessions (Stim-Non-stim) measured in 6 of the *Donor rats* (all animals A1-non-stim).

A major factor that relates to the above demonstration of enhanced memory in the *donor* as well as *delay-naïve recipient rats*, is the actual neural basis for the enhancement invoked by delivery of MIMO SR CA1 stimulation and also the type of changes that occur under normal conditions related to improved vs. impaired performance in the same subjects. The influence of administered MIMO CA1 stimulation on synaptic connectivity during the session was assessed using local field potentials (LFPs) recorded from each of the 8 CA1 locations generated by stimulating a single electrode location in CA3 on the same array (Figure 6A) with min-to-max voltage ranges. For these LFPs, it was possible to assess changes in identified voltage LFP components related to excitatory and inhibitory synaptic inputs (Hampson et al., 1989; Truccolo et al., 2002; Leung, 2011). CA1 LFPs were assessed before and after sessions in which MIMO stimulation was delivered and facilitated performance vs. sessions in which no stimulation was delivered. The most effective method of assessing such changes was to characterize differences in LFP waveforms by subtracting pre-session LFPs from post-session LFPs and comparing voltages (Post-Pre_diff_) in 10 ms segments as shown in Figure 6B. The resulting Post-Pre_diff_ waveforms reflect changes in particular components of the LFP generated from the same CA3 stimulation location related to both excitatory and inhibitory input over the same range of voltages, before and after behavioral sessions in which SR stimulation was, or was not, delivered.

Figure 6B shows the average of Post-Pre_diff_ LFP waveforms for a single trained animal following SR stimulation (blue) vs. non-stimulation (red) sessions. It is clear that the LFP changes were related directly to both excitatory (40–80 ms) and inhibitory (90–175 ms) components of well-characterized LFPs recorded from the cell layer at each of the eight CA1 locations (Leung, 2011). The lack of as much change in Post-Pre_diff_ LFP measures for non-stimulation (red curve) vs. SR stimulation (blue curve) sessions in Figure 6B reflects the increase in excitatory synaptic input to the same CA1 locations that received strong code SR CA1 stimulation patterns and facilitated performance during the behavioral sessions in the same animal. To demonstrate a more general feature of this effect, the dotted curve in Figure 6B reflects a further difference of the LFP Post-Pre_diff_ values in terms of subtracted Stim session Post-Pre_diff_ LFP waveforms from similar Non-stim session waveforms, i.e., Diff_stim_—Diff_non−stim_ LFP values. Thus, the dotted curve in Figure 6B reflects the average differences in LFPs across 5 individual animals calculated in the same manner as the two Post-Pre_diff_ LFP difference curves (red and blue) but using Post-Pre_diff_ LFPs instead to provide the resulting dotted curve average difference of LFP waveform for SR stim vs. non-stim sessions (i.e., Diff_stim_—Diff_non−stim_). Since this average difference (Diff_stim_—Diff_non−stim_) across all animals (*n* = 5) reflects alteration in the same LFP components as shown for individual waveforms in a single animal (red and blue Post-Pre_diff_ LFPs), it is clear that synaptic processes mediating CA3-to-CA1 transmission were increased by MIMO stimulation delivery during the DNMS sessions in which performance within and between animals was facilitated (Figures 4, 5).

## Discussion

### Donor/recipient recovery of hippocampal memory: a model for application to memory deficits

The above findings provide highly significant evidence that functional working memory can be enhanced by delivery of *donor rat* MIMO CA1 patterned electrical stimulation to the CA1 field in the hippocampus of *recipient rats.* This shows that information encoded by individual neural events in naïve *recipient rats* can be effectively altered by substitution of *donor rat* MIMO derived electrical stimulus patterns in the same manner as demonstrated in prior studies in which effective stimulus trains were generated by, and delivered to, the same animal (Berger et al., 2011; Hampson et al., 2012a). The demonstration of improved task performance in naïve *recipient rats* (Figures 4, 5), verifies that *donor rat* SR CA1 stimulation was capable of inducing the type of encoding process necessary when facilitated retention of information was required because of interposed unfamiliar delays of variable duration (Figure 6). The fact that MIMO stimulation can approximate normal ensemble firing involved in the encoding and retrieval of task-relevant information is consistent with other recent findings investigating relationships between multineuron firing in cortical ensembles and behavioral task requirements (Ross and Eichenbaum, 2006; Komorowski et al., 2009; Smith et al., 2009; Rouse et al., 2011). However, the demonstration that the patterns marked as effective and generated online in one animal, could be transferred via temporally matched electrical stimulation of similar CA1 regions in naïve *recipient* animals exposed to the same task contingencies, has not been shown previously. Although consistent in some ways with a recent “brain-to-brain transfer” experiment (Pais-Viera et al., 2013) in which sensorimotor cortical signals were used to influence behavioral choice in the recipient rodent, the results presented here differ significantly because in that experiment, stimulation was delivered at the time of the behavioral response, whereas in our study, the stimulation corresponded to the encoding phase of the task (SR) and was delivered up to 16 s prior to the behavioral response, confirming transfer of a *memory* code, and not simply induction of a motor response. In addition, the lack of enhancement or transfer when several control procedures were employed in the above memory transfer paradigm; i.e., temporal relation to SR, reduced stimulation intensity, closed-loop dependence, etc. (Figures 1–5), strongly supports the specificity of the transference of *donor rat* MIMO model derived SR information for hippocampal function to naïve *recipient rats* for task-relevant performance.

These results provide important insight for extending *donor/recipient* procedures to functions performed by other brain regions and other behavioral endpoints as shown recently (Pais-Viera et al., 2013), and eventually to similar circumstances involving humans (Boettiger and D'Esposito, 2005; Smith et al., 2009; Hasson et al., 2012). Once fabricated into a neural prosthesis for *recipients* this unique technology could (1) immediately enhance task-specific performance, (2) repair damaged or impaired task-dependent brain circuitry, and possibly even, (3) provide neural encoding of task-relevant information without prior training. The long history of investigation with hippocampal recording in the behavioral context employed, and prior collaboration perfecting application of the MIMO model to these recordings (Hampson et al., 2008, 2012a; Berger et al., 2012; Marmarelis et al., 2013; Song et al., 2013), as well as recent applications to the non-human primate hippocampus (Hampson et al., 2013) and prefrontal cortex (Hampson et al., 2012c; Opris et al., 2012), provided the insight necessary to extrapolate how *donor/recipient* memory transference could occur as demonstrated here. However, the fact that transferred patterns of electrical brain stimulation have significant functional impact and are capable of modifying performance via strategic online delivery provides another demonstration of *donor-recipient* brain compatibility (Pais-Viera et al., 2013), but this application to hippocampus for improving memory is the first demonstration specific to brain cognitive function. Such results not only provide important new insight into how hippocampal circuits can be operated to process memory-dependent information via external control, but also provide a basis for extending and/or perfecting similar *donor/recipient* type devices (Jarosiewicz et al., 2008; Venkatraman and Carmena, 2011; Hasson et al., 2012) to enhance and/or replace memory deficiencies in humans.

## Conflict of interest statement

The authors declare that the research was conducted in the absence of any commercial or financial relationships that could be construed as a potential conflict of interest.
