# Supplementary material for: Donor/recipient enhancement of memory in rat hippocampus
Source: Front Syst Neurosci. 2013 Dec 26;7:120. doi: 10.3389/fnsys.2013.00120 (PMC3872745; doi:10.3389/fnsys.2013.00120)
Supplement: Supplementary file 1 [file Presentation1.PDF]

Supplemental Online Material

Methods

Subjects and Training

Animals: Male, Long-Evans rats (Harlan) aged 4-6 months were individually housed allowed free access to food and water restricted to maintain 85% of ad libitum body weight, monitored daily with access restricted until after testing. All animal protocols were approved by the Wake Forest University Institutional Animal Care and Use Committee, Association for Assessment and Accreditation of Laboratory Animal Care and the National Institute of Health Guide for the Care and Use of Laboratory Animals (8th Edition, 2011).

Apparatus: The behavioral testing apparatus for the delayed nonmatch to sample (DNMS) task is the same as reported in other studies from this laboratory [1-3] and consisted of a 43 × 43 × 50 cm Plexiglass chamber with two retractable levers (left and right) positioned on either side of a water trough on the front panel and a nose-poke device mounted in the center of the opposite back panel. A cue light was positioned immediately above the nose-poke device. A video camera was mounted above and the entire chamber was housed inside a commercially built sound-attenuated cubicle.

Behavioral training procedure: The DNMS task consisted of three main phases: sample, delay and nonmatch. At the initiation of a trial, either the left or right lever was extended randomly with 50% overall probability and the animal was required to press this lever (Sample Response, SR) to initiate the delay phase of the task signaled by the illumination of a cue light over the nose-poke device in the rear panel after the Sample lever was immediately retracted. The animal was required to nose-poke in the photocell device at least once during the variable duration (1-30s) delay interval with each delay presented randomly on each trial during a session. The last nose poke (LNP) after the delay timed out turned off the cue light and extended both levers on the front panel, signaling the onset of the Nonmatch phase. In the Nonmatch phase, to make a correct response, the animal was required to press the lever opposite that pressed during the Sample phase (Nonmatch Response or NR) to obtain a water reward (Reinforcement). After the NR the levers were retracted for a 10.0s intertrial interval ITI before the next trial. A response on the same lever as the SR (Match Response) constituted an "error" with no water delivery and the chamber house lights were turned off for 5.0s after which the house lights were turned back on and the next trial presented 5.0 s later. Individual performance was assessed as % correct NRs with respect to the total #trials (100-150) with delays of 1-30s presented over a 1-2 hr session.

Surgery

Hippocampal Electrode Arrays: All surgical procedures conformed to National Institutes of Health and Association for Assessment and Accreditation of Laboratory Animal Care guidelines, and were performed in a rodent surgical facility approved by the Wake Forest University Institutional Animal Care and Use Committee. Following training to criterion performance levels in the DNMS task animals were anesthetized with ketamine (100 mg/kg) and xylazine (10 mg/kg) and placed in a stereotaxic frame. Craniotomies (5mm-diameter) were performed bilaterally over the dorsal hippocampus to provide for two pairs of array electrodes (Neurolinc, New York, NY), consisting of two rows of 8 stainless steel wires (diameter: 20µm each) positioned such that the geometric center of each electrode array was centered at co-ordinates 3.4 mm posterior to Bregma and 3.0 mm lateral (right or left) to midline [4]. The array was designed such that the distance between two adjacent electrodes within a row was 200 µm and between

rows was 400 µm apart to conform to the locations of the respective CA3 and CA1 cell layers. The longitudinal axis of the array was angled 30° to the midline during implantation to conform to the anatomic position of hippocampus, with posterior electrode sites more lateral than anterior sites. Each array was lowered in 25 -100 µm steps to a depth of 3.0 - 4.0 mm from the cortical surface for the longer electrodes positioned in the CA3 cell layer, leaving the shorter CA1 electrodes1.2 mm higher in the CA1 layer. Neural activity recorded from the electrodes was monitored during surgery to ensure placement in the appropriate hippocampal cell layers. After placement of the array the cranium was sealed with bone wax and dental cement and the animals received buprenorphine (0.01–0.05 mg/kg) for pain relief over the next 4-6 hrs. The scalp wound was treated periodically with Neosporin antibiotic and systemic injections of penicillin G (300,000 U, intramuscular) were given to prevent infection. Animals were allowed to recover from surgery for at least 1 week before continuing behavioral testing [5-7].

Intrahippocampal Cannula and Osmotic Minipumps: During array implant surgery some animals were also fitted with intrahippocampal infusion cannula attached to minipumps (8,9). Cannula consisted of stainless steel hypodermic tubing (26 gauge) connected via 24 gauge silicone tubing to an osmotic minipump implanted subcutaneously below the neck. The tip of each cannula was directed to coordinates: 4.0 mm posterior to bregma, 4.0 mm lateral to midline; angled to place the cannula tip within the dorsal CA3 region at a depth of 4.0-4.2 mm. The unilaterally implanted cannula were secured in position with dental cement and attached to an Alzet® osmotic minipump (Model 2004, Durect Corp., CA) containing either saline vehicle or MK801. All animals were given 5 to 7 days to recover prior to retraining and testing. Minipumps were filled with physiological saline (vehicle) at the time of surgery and after 2-4 weeks of control testing with vehicle minipump contents were changed to MK801 in saline vehicle. For this procedure (10) animals were anesthetized with ketamine (100 mg/kg) and xylazine (10 mg/kg), the spent minipump removed by cutting the silicone tubing and the drug-filled minipump attached to the tube and replaced in the same subcutaneous pouch. Animals were allowed to recover for 2 days prior to resumption of testing.

Electrophysiology: Multi-neuron Recording

Acquisition of Neuronal Data: For electrophysiological monitoring animals were connected by cable to the recording apparatus via a 32-channel headstage and harness attached to a 40-channel slip-ring commutator (Crist Instruments, Hagerstown, MD) to allow free-motion of the animal in the behavioral testing chamber (7,11-13). Single neuron action potentials (spikes) were isolated by time-amplitude window discrimination and computer-identified individual waveform characteristics using a multi-neuron acquisition (MAP) processor (Plexon Inc., Dallas, TX, USA). Single neuron spikes were recorded daily using waveform and firing characteristics within the task (perievent histograms) for each of the DNMS events (SR, LNP & NR). Only isolated spike waveforms exhibiting firing rates consistent with CA1 and CA3 principal cells (i.e. 0.5-5.0 Hz baseline firing rate) and stable behavioral correlates across sessions were included in the analysis [7-10]. Final ensembles used to analyze neural encoding of DNMS events were comprised of activity from 15-30 single neurons, each recorded from a separate electrode location on the bilateral arrays.

Nonlinear Systems Analysis: Multi Input, Multi Output (MIMO) Model: A general, Volterra kernel-based strategy for modeling MIMO nonlinear dynamics underlying spike train-to-spike train transformations between CA3 and CA1 was established to predict output patterns of CA1 firing pattern from input patterns of CA3 neural activity [11-15]. In this approach, the modeling of spatio-temporal pattern transformations from an upstream hippocampal brain region, e.g., CA3, to a downstream hippocampal brain region, e.g.,

CA1, is formulated as the identification of an MIMO system that can be decomposed into a series of multiple-input, single-output (MISO) subsystems with physiologically identifiable structure that can be expressed by the following equations:

$$w = u(k,x) + a(h,y) + \varepsilon(\sigma), \quad y = \begin{cases} 0 & \text{when } w < \theta \\ 1 & \text{when } w \geq \theta \end{cases} \qquad \text{(Eq. 1)}$$

The variable xi represents input spike trains; yi represents output spike trains. The hidden variable w represents the pre-threshold membrane potential of the output neurons. It is equal to the summation of three components, i.e., synaptic potential u caused by input spike trains, the output spike-triggered after-potential a, and a Gaussian white noise input ε with standard deviation σ. The noise term models both intrinsic noise of the output neuron and the contribution of unobserved inputs. When w exceeds threshold, θ, an output spike is generated and a feedback after-potential (a) is triggered and then added to w. Feedforward kernels k describe the transformation from x to u. The feedback kernel, h, describes the transformation from y to a. u can be expressed as a Volterra functional series of x, as in

$$\begin{aligned} u(t) = k_0 &+ \sum_{n=1}^{N}\sum_{\tau=0}^{M_k} k_1^{(n)}(\tau)x_n(t-\tau) + \sum_{n=1}^{N}\sum_{\tau_1=0}^{M_k}\sum_{\tau_2=0}^{M_k} k_{2s}^{(n)}(\tau_1,\tau_2)x_n(t-\tau_1)x_n(t-\tau_2) \\ &+ \sum_{n_1=1}^{N}\sum_{n_2=1}^{n_1-1}\sum_{\tau_1=0}^{M_k}\sum_{\tau_2=0}^{M_k} k_{2x}^{(n_1,n_2)}(\tau_1,\tau_2)x_{n_1}(t-\tau_1)x_{n_2}(t-\tau_2) \\ &+ \sum_{n=1}^{N}\sum_{\tau_1=0}^{M_k}\sum_{\tau_2=0}^{M_k}\sum_{\tau_3=0}^{M_k} k_{3s}^{(n)}(\tau_1,\tau_2,\tau_3)x_n(t-\tau_1)x_n(t-\tau_2)x_n(t-\tau_3) + \ldots \end{aligned} \qquad \text{(Eq. 2)}$$

The zeroth order kernel, k0, is the value of u when the input is absent. First order kernels, k1(n), describe the linear relation between the nth input xn and u. Second and third order self-kernels, k2(n), and k3(n), describe the 2nd and 3rd order nonlinear relation between the nth input xn and u, respectively. Second order cross-kernels k2(n1,n2), describe the 2nd order nonlinear interactions between each unique pair of inputs (xn1 and xn2) as they affect u. N is the number of inputs. Mk denotes the memory length of the feedforward process. The feedback variable a can be expressed as:

$$a(t) = \sum_{\tau=1}^{M_h} h(\tau)y(t-\tau) \qquad \text{(Eq. 3)}$$

where h is the linear feedback kernel. Mh is the memory of the feedback process. In total, then, the model describes how temporal patterns of third-order (i.e., the effects of triplets) for each input, and second-order (i.e., the effects of pairs) for any of two interacting inputs, affect each output, taking into account differing noise level and output spike-triggered feedback (the latter due to circuitry and/or membrane biophysics), and neuron-specific differences in thresholds. In order to reduce the number of open parameters to be estimated, both k and h are expanded with orthonormal Laguerre basis functions [16]. Due to the Gaussian noise term and the threshold, this model can be considered a special case of the Generalized Laguerre-Volterra Model (GLVM) which employs a probit link function [11;17]. All model parameters, i.e., feedforward Volterra kernel k and feedback Volterra kernel h can be estimated using an iterative re-weighted least-squares method [18]. Noise deviation σ and threshold θ are redundant variables and thus can be indirectly obtained through variable transformation [13;17]. Due to the

stochastic nature of the system, estimated models are validated using an out-of-sample Kolmogorov-Smirnov test based on the time-rescaling theorem [19].

Prediction of Strong and Weak Neural Encoding Using MIMO Model: MIMO models were computed from 3-5 consecutive DNMS sessions which recorded a minimum of 8 CA3 and 8 CA1 neurons. MIMO models which accurately predicted CA1 (output) spike trains based on CA3 (input) spike trains, and required at least second-order self-kernels (k2s) to sufficiently capture the CA3-CA1 nonlinear dynamics were retained for further testing. Coefficients of the MIMO models which met criteria for CA1 spike train prediction were then tested using 5 DNMS sessions not used to derive the model. Predicted CA1 firing patterns were synchronized to Sample Response DNMS events in the recorded sessions, and used to construct peri-event histograms (PEHs) of predicted CA1 neural firing in the Sample phase (i.e. ± 1.5 s around Sample Response). Predicted CA1 PEHs were further sorted according to correct vs. error behavioral outcome for each trial, as well as trial delay. "Strong" SR codes were then identified as the mean predicted CA1 firing pattern on trials that were correct at the longest delays (i.e. correct trials at 21-30 s delays), while "weak" SR codes were identified as the mean predicted CA1 firing pattern on trials that were errors at the shortest delays (i.e. error trials at 1-10 s delays). Single trials were identified as consistent with strong and weak SR codes if their mean CA1 firing patterns corresponded to the mean predicted strong and weak code PEHs. strong SR code trials predicted by the MIMO model were utilized for Closed Loop and electrical stimulation procedures.

Closed Loop Procedures

Hippocampal Neuro-Behavioral Closed Loop Paradigm: Predictions of behavioral outcome by either the linear or MIMO nonlinear analyses were incorporated into a “Closed Loop” feedback paradigm implemented in the DNMS task. The procedure consisted of altering the duration (in seconds) of the Delay interval between the Sample and Nonmatch phase as a function of the SR code “strength” (strong or weak) determined on the same trial. If ensemble SR codes were not strong or weak the closed loop manipulation was not implemented and the trial conformed to whatever delay was programmed to occur randomly as in normal training sessions. Closed Loop trials consisted of the following manipulations:1) the delay interval was shortened to 10 s for weak SR code trials; or 2) extended to one of three randomly-selected longer durations: 40, 50, or 60 s when a strong SR code was detected. Closed Loop trials were interspersed randomly on approximately 25-30% of total trials within a session, and sessions employing the procedure were also randomly interspersed among normal sessions in which no Closed Loop trials were present. The number of Closed Loop sessions per animal ranged from 12-49.

Stimulation: Closed Loop: A custom built 16 channel stimulator (Triangle BioSystems, Inc, Durham, NC) was employed to deliver patterned of electrical stimulation pulses to CA1 electrodes in bilateral hippocampal arrays. The stimulator delivered digital-to-analog (D/A) converted biphasic output pulses to the 8 pairs of CA1 electrodes. Each D/A output channel delivered one-half of a symmetric biphasic stimulation pulse to a single pair of adjacent wires in CA1 allowing bipolar stimulation that was isolated from other electrodes in the same array. Biphasic stimulator pulses (1.0 ms duration) were electronically gated to produce square constant voltage outputs in the range of 0.1 to 15 V (20-100μA) in 0.05 volt increments with minimum interpulse intervals of 0.5 ms on a given channel. Parameters typically employed on a single channel were: biphasic, 1.0-4.0V p-p, 1.0ms, ≤ 10.0Hz. On Closed Loop trials MIMO stimulation consisted of mean nonlinear MIMO pulse patterns of predicted CA1 firing on strong SR code trials via MIMO analysis of CA3 input firing on the same trial. Stimulation patterns were 3.0 s in duration

consisting of 8 channels of bilateral CA1 pulses delivered synchronous with SR occurrence, or, shifted to occur >3.0s after completion of the SR for control purposes.

Donor/Recipient Rat Stimulation Paradigm: This procedure utilized synchronized simultaneous testing of two animals, one rat fully trained in the DNMS task to criterion performance on trials with delays of 1-30s (Donor Rat) and the other (Recipient Rat) trained only to a stage in which Nonmatch responses were performed immediately following the nosepoke with no delay interval interposed between the Sample and Nonmatch phases. Both animals were tested simultaneously in adjacent chambers with trial onset synchronized with respect to Sample lever presentation. MIMO strong SR code CA1 stimulation patterns were computed online from CA3 firing recorded from the Donor Rat and delivered to the Recipient Rat when the latter performed the SR. On Recipient Rat stimulation trials, delays (8-16s) were interposed following the SR by not extinguishing the nosepoke cue light nor presenting the two levers in the Nonmatch phase until after the interposed delay timed out. Performance was evaluated within the same session on the basis of mean % correct responses on stimulation trials with extended delays compared to performance on trials with the same interposed delays but no stimulation delivered.

Supplemental References

[1] S. A. Deadwyler, T. Bunn, and R. E. Hampson, "Hippocampal ensemble activity during spatial delayed-nonmatch-to-sample performance in rats," J. Neurosci., vol. 16, pp. 354-372, 1996.

[2] R. E. Hampson, L. E. Jarrard, and S. A. Deadwyler, "Effects of ibotenate hippocampal and extrahippocampal destruction on delayed-match and -nonmatch-to-sample behavior in rats," Journal of Neuroscience, vol. 19, pp. 1492-1507, Feb.1999.

[3] S. A. Deadwyler and R. E. Hampson, "Differential but complementary mnemonic functions of the hippocampus and subiculum," Neuron, vol. 42, pp. 465-476, 2004.

[4] G. Paxinos and C. Watson, The Rat Brain in Stereotaxic Coordinates, 3 ed. San Diego: Academic Press, 1997.

[5] S. A. Deadwyler and R. E. Hampson, "Endocannabinoids modulate encoding of sequential memory in the rat hippocampus," Psychopharmacology (Berl), vol. 198, pp. 577-586, Jan.2008.

[6] R. E. Hampson, J. D. Simeral, and S. A. Deadwyler, "Neural population recording in behaving animals: Constituents of the neural code for behavior," in Neural Population Encoding. C. Holscher and M. H. Munk, Eds. Cambridge, UK: Cambridge University Press, 2008, pp. 74-94.

[7] A. V. Goonawardena, L. Robinson, G. Riedel, and R. E. Hampson, "Recruitment of hippocampal neurons to encode behavioral events in the rat: Alterations in cognitive demand and cannabinoid exposure," Hippocampus, 2009.

[8] R. E. Hampson, J. D. Simeral, and S. A. Deadwyler, "Cognitive processes in replacement brain parts: A code for all reasons," in Toward Replacement Parts for the Brain. Implantable Biomimetic Electronics as Neural Prosthesis. T. W. Berger and D. L. Glanzman, Eds. Cambridge, MA: MIT Press, 2005, pp. 111-128.

[9] S. A. Deadwyler and R. E. Hampson, "Temporal coupling between subicular and hippocampal neurons underlies retention of trial-specific events," Behav. Brain Res., vol. 174, pp. 272-280, Nov.2006.

[10] S. A. Deadwyler, A. V. Goonawardena, and R. E. Hampson, "Short-term memory is modulated by the spontaneous release of endocannabinoids: evidence from hippocampal population codes," Behav. Pharmacol., vol. 18, pp. 571-580, Sept.2007.

[11] D. Song, R. H. M. Chan, V. Z. Marmarelis, R. E. Hampson, S. A. Deadwyler, and T. W. Berger, "Generalized Volterra model for spike train transformations in the hippocampus," 2008, p. Program No. 695.3.

[12] T. P. Zanos, S. H. Courellis, T. W. Berger, R. E. Hampson, S. A. Deadwyler, and V. Z. Marmarelis, "Nonlinear modeling of causal interrelationships in neuronal ensembles," IEEE Trans. Neural Syst. Rehabil. Eng, vol. 16, pp. 336-352, Aug.2008.

[13] D. Song, R. H. Chan, V. Z. Marmarelis, R. E. Hampson, S. A. Deadwyler, and T. W. Berger, "Nonlinear modeling of neural population dynamics for hippocampal prostheses," Neural Netw., May2009.

[14] T. P. Zanos, R. E. Hampson, S. E. Deadwyler, T. W. Berger, and V. Z. Marmarelis, "Boolean Modeling of Neural Systems with Point-Process Inputs and Outputs. Part II: Application to the Rat Hippocampus," Ann. Biomed. Eng, June2009.

[15] D. Song, R. H. Chan, V. Z. Marmarelis, R. E. Hampson, S. A. Deadwyler, and T. W. Berger, "Nonlinear dynamic modeling of spike train transformations for hippocampal-cortical prostheses," IEEE Trans. Biomed. Eng, vol. 54, pp. 1053-1066, June2007.

[16] V. Z. Marmarelis, "Identification of nonlinear biological systems using Laguerre expansions of kernels," Ann. Biomed. Eng., vol. 21, pp. 573-589, 1993.

[17] D. Song, R. H. Chan, V. Z. Marmarelis, R. E. Hampson, S. A. Deadwyler, and T. W. Berger, "Nonlinear dynamic modeling of spike train transformations for hippocampal-cortical prostheses," IEEE Trans. Biomed. Eng, vol. 54, pp. 1053-1066, June2007.

[18] W. Truccolo, U. T. Eden, M. R. Fellows, J. P. Donoghue, and E. N. Brown, "A point process framework for relating neural spiking activity to spiking history, neural ensemble, and extrinsic covariate effects," J. Neurophysiol., vol. 93, pp. 1074-1089, Feb.2005.

[19] E. N. Brown, R. Barbieri, V. Ventura, R. E. Kass, and L. M. Frank, "The time-rescaling theorem and its application to neural spike train data analysis," Neural Comput., vol. 14, pp. 325-346, Feb.2002.
